# Genetic and Epigenetic Characterization of Pulpal and Periapical Inflammation

**DOI:** 10.3389/fphys.2020.00021

**Published:** 2020-02-04

**Authors:** Ashraf F. Fouad, Asma A. Khan, Renato M. Silva, Mo K. Kang

**Affiliations:** ^1^Division of Comprehensive Oral Health, Adams School of Dentistry, University of North Carolina at Chapel Hill, Chapel Hill, NC, United States; ^2^Department of Endodontics, University of Texas Health Science Center at San Antonio, San Antonio, TX, United States; ^3^Department of Endodontics, The University of Texas Health Science Center at Houston, Houston, TX, United States; ^4^Section of Endodontics, School of Dentistry, University of California, Los Angeles, Los Angeles, CA, United States

**Keywords:** dental pulp, periapical disease, inflammatory mediators, genetic polymorphism, epigenetics

## Abstract

Pulpal and periapical diseases affect a large segment of the population. The role of microbial infections and host effector molecules in these diseases is well established. However, the interaction between host genes and environmental factors in disease susceptibility and progression is less well understood. Studies of genetic polymorphisms in disease relevant genes have suggested that individual predisposition may contribute to susceptibility to pulpal and periapical diseases. Other studies have explored the contribution of epigenetic mechanisms to these diseases. Ongoing research expands the spectrum of non-coding RNAs in pulpal disease to include viral microRNAs as well. This review summarizes recent advances in the genetic and epigenetic characterization of pulpal and periapical disease, with special emphasis on recent data that address the pathogenesis of irreversible pulpal pathosis and apical periodontitis. Specifically, proinflammatory and anti-inflammatory gene expression and gene polymorphism, as well as recent data on DNA methylation and microRNAs are reviewed. Improved understanding of these mechanisms may aid in disease prevention as well as in improved treatment outcomes.

## Introduction

Pulpal and periapical diseases involve a large segment of the population. This common occurrence is related to the high prevalence of their principal etiologic factors, namely dental caries and traumatic injuries. Moreover, the cumulative burden of the effects of repeated failure of restorations, secondary disease and tooth loss further increase the prevalence and significance of endodontic pathosis. Dental caries continues to be among the most prevalent diseases that affects humans, especially among younger individuals. For example, in the United States caries prevalence among 2–19 year-old individuals is 46% (Fleming and Afful, 2018). Dental caries is associated with inflammation in the dental pulp, even at its earliest stages (Brannstrom and Lind, 1965). Fortunately, most of the early phases of caries cause reversible pulpitis, which heals following the restoration of the tooth. However, because caries is mostly asymptomatic, many cases progress to irreversible pulpitis. This condition may or may not be associated with significant pain and if untreated would progress to pulp necrosis.

The conservative management of pulpitis, which aims to preserve the vitality of this tissue, is predicated on the accurate diagnosis of the condition of the dental pulp. The clinical methods involved in making this diagnosis vary widely, are not standardized and have not been clinically validated. Traditionally, carious pulp exposure was deemed a sign of irreversible pulp pathosis, regardless of symptoms. However, in the last decade, vital pulp therapy procedures have gained considerable popularity, with the realization that a carious pulp exposure does not automatically denote irreversible pulpitis (Bogen et al., 2008), that the pain from symptomatic pulpitis can be effectively managed with pulpotomy procedures (Eren et al., 2017; Galani et al., 2017), and that tricalcium silicates are effective materials for preserving the long-term vitality of the pulp (Schwendicke et al., 2016; Elmsmari et al., 2019; Li et al., 2019).

However, pain is not a constant finding with pulpitis; in fact, it is estimated that about 40% of all cases with pulpitis progress to pulp necrosis without symptoms (Michaelson and Holland, 2002). Moreover, most of the available evidence on outcomes of vital pulp therapy is in the 1–2 years post-operatively. Therefore, better tools for diagnosis of the status of the pulp and better prediction of pulp survival following treatment are highly desirable.

**FIGURE 1:**
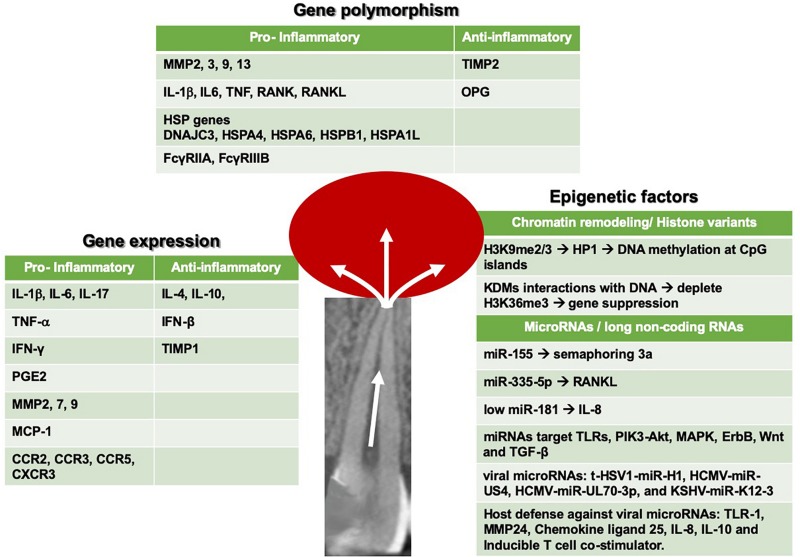
Summary of seminal gene expression, gene polymorphism and epigenetic mediators, and pathways that have been recently reported in pulp and periapical inflammation.

In this review, recent data on the genetic and epigenetic effectors of pulpal and periapical inflammatory responses that lead to irreversible pathosis will be reviewed. This review is not intended to be an exhaustive review of the subject of pulpal and periapical pathosis, which is better detailed in other sources (Fouad and Huang, 2019). Rather, the focus will be on recent findings related to the most prominent effectors and disease pathways of this pathological process, as shown in Figure 1.

Research on understanding the molecular changes in the pulp that lead to true irreversible pathosis could provide valuable insights, and future tools to aid in diagnosis and treatment planning of these conditions. Sequelae of pulp necrosis include apical periodontitis (AP) which manifests as, pain, swelling, purulent drainage, bone and/or tooth loss. Occasionally the infection spreads to distant sites, causing significant morbidity or even mortality. Inflammation and infection of the periapical tissues ultimately leads to bone resorption, which results from enhanced osteoclastogenesis induced through receptor activator of nuclear factor kappa B (RANK) ligand and accumulation of pro-inflammatory cytokines, e.g., IL-1β, TNF-α, prostaglandin E2 and IL-17 (Zou and Bar-Shavit, 2002; Ritchlin et al., 2003; Suda et al., 2004; AlShwaimi et al., 2013; Rechenberg et al., 2014). RANK ligand is presented by osteoblasts and binds to the RANK receptor on the osteoclast precursors, which then triggers their differentiation into mature osteoclasts (Yasuda et al., 1998; Udagawa et al., 1999). Understanding the kinetics of bone resorption in AP would allow the development of diagnostic tools that could differentiate actively expanding lesions from those that are inactive or healing. In patient samples of periapical granulomatous lesions, there was elevation of RANK ligand compared to normal counterparts, and infected root apices contained higher level of pro-inflammatory cytokines that support recruitment of inflammatory cells (Vernal et al., 2006; Rechenberg et al., 2014). Several key studies demonstrated pro-inflammatory nature of periapical granulomas; elevation of IL-1β, IL-6, TNF-α and IL-17 was documented in symptomatic AP compared with asymptomatic AP in patients (Oseko et al., 2009; Jakovljevic et al., 2015). Periapical lesions also revealed increased level of chemokines, e.g., MCP-1 and CCR2, and chemokine receptors, e.g., CCR3, CCR5, and CXCR3, resulting in recruitment of pro-inflammatory cells to the periapex (Kabashima et al., 2001; Cavalla et al., 2013; Liu et al., 2014). Hence, RANK-mediated osteoclastogenesis, led by cascade of pro-inflammatory signaling, would likely play crucial roles in the periapical bone destruction in AP.

While the above studies demonstrate the association between pro-inflammatory signaling and periapical bone destruction in AP, a series of genetic studies revealed functional roles of various cytokines in AP. For instance, a recent study demonstrated increased apical bone loss after pulpal exposure in IL-17 receptor antagonist (IL-17RA) knockout (KO) mice with increased level of neutrophils and macrophages and increased bone resorption compared with the wild-type (WT) littermates, suggesting an immune-modulatory effects of IL-17RA signaling in AP (AlShwaimi et al., 2013). Likewise, Th2-type cytokines, e.g., IL-4 and IL-10, were assessed for their functional effects in periapical bone destruction in a genetic study, which revealed the suppressive role of IL-10, but not IL-4, on infection-induced periapical bone loss (Sasaki et al., 2000). These types of loss-of-function studies, combined with expression/associative studies, have led to accumulation of knowledge in the functional roles of diverse array of “effector” molecules of periapical inflammation, such as the pro-inflammatory cytokines and other inflammatory mediators, e.g., nitric oxide synthase (iNOS) and prostaglandins, in the pathogenesis of AP (McNicholas et al., 1991; Silva et al., 2011). However, little is known about the mechanisms that regulate the levels of such effector molecules of AP and their differential contributions to pathogenesis and healing. Therefore, understanding the molecular mediators of AP at the proteomic, genomic and epigenetic levels, particularly in asymptomatic conditions, and in cases with persistent disease is also critical for planning care and determining outcomes of treatment of this disease.

## Genetic Susceptibility to Pulpitis and Apical Periodontitis

Evidence suggests that dental caries, pulpal and periapical inflammation may be related to individual predisposition to develop the disease (Aminoshariae and Kulild, 2015). The human genome consists of approximately 60,000 genes, of which 20,000 are known protein-coding genes (Venter et al., 2001; Karki et al., 2015). A single nucleotide polymorphism is a change in one nucleotide in an individual’s DNA sequence and occurs approximately 1% in the population (Brookes, 1999). These genetic variations in each individual may contribute to different disease presentations and/or treatment outcomes, therefore two general questions about differential individual response to bacterial challenge have been the field of several studies:

(1) Why some individuals with a deep carious lesion readily develop pulpal and periapical pathologies while others with similar carious lesions resist the development of pulpitis and AP?

(2) Why some individuals present with persistent AP or delayed healing after adequate endodontic treatment?

To address these questions, genetic association studies of individuals with the aforementioned characteristics have assessed the underlying etiology of pulpal and periapical diseases. To date, a few disease-relevant genes have been suggested as candidates in association studies of pulpal and periapical diseases. The most promising candidate gene families are discussed in detail below.

## Matrix Metalloproteinases (MMPs) and Tissue Inhibitors of Metalloproteinases (TIMPs)

Matrix metalloproteinases are secreted as proenzyme forms requiring extracellular activation. They are regulated by their endogenously secreted inhibitors called TIMPs (Birkedal-Hansen et al., 1993). They are responsible for tissue remodeling and appear to have an influential role in dental caries, pulpitis and AP development (Tannure et al., 2012; Letra et al., 2013).

Matrix metalloproteinases participate in the bone organic matrix remodeling process (Everts et al., 1992), and their expression may be stimulated by interleukin-1 and -6 (Jakovljevic et al., 2015). MMP 2, 7 and 9 in particular appear to have a predominant role in chronic apical abscesses cases in which ongoing tissue destruction is observed (Letra et al., 2013). On the other hand, TIMPs may inhibit the activity of some MMPs (Sternlicht and Werb, 2001). *TIMP-1* may also act as an inhibitor of bone resorption (Sobue et al., 2001). MMP and TIMP may be directly involved with AP development and activity. Recently, *TIMP1* mRNA was found to be highly expressed in human periapical granulomas, particularly those with molecular characteristics of an inactive lesion (Letra et al., 2013).

Matrix metalloproteinases and TIMPs were reported as associated with dental caries development. Single nucleotide polymorphisms (SNPs) in *MMP2* (rs243865), *MMP9* (rs17576), *MMP13* (rs2252070), and *TIMP2* (rs7501477) were investigated for their association with caries in 505 subjects, 212 caries-free subjects and 293 subjects presenting with dental caries. Allele frequencies for *MMP2*, *MMP13* and *TIMP2* polymorphisms were significantly different between individuals with or without dental caries. Moreover, mutant allele carriers for *MMP13* demonstrated a significantly decreased risk for dental caries, even when adjusting the analyses considering candidate genes, type of dentition and dietary factors, suggesting that genetic polymorphisms in *MMP13* may contribute to individual caries susceptibility (Tannure et al., 2012).

Single nucleotide polymorphisms in *MMP2* and *MMP3* genes have also been shown to contribute to the development of AP (Menezes-Silva et al., 2012). Overall, variations in five MMP genes and one TIMP gene (totaling 16 variants) were associated with pulpal inflammation and AP development in teeth affected by extensive caries lesions in 268 individuals (158 control individuals presenting carious lesions and no AP, and 110 individuals presenting carious lesions and AP). Two SNPs in *MMP3* (rs679620 and rs639752), comprising a missense mutation and an intronic single base substitution, respectively, were significantly associated with cases with extensive dental caries and presence of AP (*P* = 0.004 and *P* = 0.03, respectively). The associated missense mutation in *MMP3* (rs679620) results in a lysine-to-glutamine substitution in the final protein structure, and is predicted to have functional effects as it has been associated with increased MMP3 expression (Ye et al., 1996). It is possible that this variant could be used as a potential target for dental caries progression into dentin and its downstream effects on the pulp, such as pulp necrosis and ultimately, AP. In addition, altered transmission of *MMP2* SNP alleles was detected in cases with deep caries and presence of AP (0.00004 ≤ *P* ≤ 0.002). Collectively, these results suggest that MMPs may be associated with AP development and progression caused by untreated deep dental caries. Further, genetic polymorphisms in MMP and TIMP genes may interfere with apical tissue destruction and remodeling resulting in active AP in teeth affected by dental caries (Menezes-Silva et al., 2012).

## Pro-Inflammatory Cytokines

Cytokines may be directly related to healing and host response to infection, inflammation, and trauma. Considering the impact of cytokines on host tissues, pro-inflammatory cytokines generally act as catabolic factors and mediate disease-associated tissue destruction, whereas anti-inflammatory cytokines usually function to reduce inflammation and promote healing (Dinarello, 2000).

Several cytokines, such as tumor necrosis factor-α (*TNF-*α), interleukin-1 (*IL-1*), and interleukin-6 (*IL-6*), are involved in soft tissue degradation and bone resorption directly or indirectly promoting osteoclastogenesis, whereas *IL-4*, and interferon-β (*IFN-*β) favor bone formation by inhibiting osteoclastogenesis (Birkedal-Hansen et al., 1993; Baker-LePain et al., 2011). Interferon-gamma (IFN-y) is released by activated T-lymphocytes while *TNF-*α is released by both lymphocytes and macrophages. These cell types are frequently located at sites of tissue damage due to chronic infection or autoimmune disease (Dinarello, 2000; Baker-LePain et al., 2011).

Both genetic and biological interactions between cytokines and MMPs have been reported in the literature. IL-1β plays an essential role in both acute and chronic inflammatory processes (Ito et al., 1996). It is secreted from activated macrophages and other cell types. Cytokines can also modulate MMPs transcription. Cells stimulated with IL-1 may increase MMPs production and contribute to wound healing and tissue degradation (Ito et al., 1996). MMPs (particularly MMP-1, -2, -3 and -9) are able to process the recombinant IL-1β precursor molecule into biologically active forms and thereby upregulate IL-1β activity, or degrade IL-1β into inactive fragments thus downregulate its activity (Ito et al., 1996).

Cytokines are established players in pulpal and periapical tissue destruction (Araujo-Pires et al., 2014). Polymorphisms in cytokine genes were reported to increase risk to developing AP in response to dental carious lesions (Dill et al., 2015). In a previous study, 316 cases [136 with extensive dental caries and AP (cases) and 180 with extensive dental caries but without AP (controls)] were tested for association with polymorphisms in cytokine genes. SNPs in *IL1B, IL6, TNF, RANK, RANKL*, and *OPG* genes were genotyped, and significant association was found between an intronic polymorphism in *IL1B* (rs1143643) and cases of deep caries and AP. Altered transmission of *IL1B* haplotypes was also observed in association with AP. Furthermore, the expression of *IL1B* was reported as markedly higher in human periapical granuloma tissue samples when compared to healthy periodontal ligament tissues (Ito et al., 1996).

## Heat Shock Proteins (HSPs)

Heat shock proteins are a family of proteins often referred to as molecular chaperones with essential roles in protein synthesis, transport, and folding. HSPs are subdivided based on their molecular weight and are induced by stress signals such as fever, hypoxia, infectious agents, and inflammatory mediators (Morimoto, 1993, 1998). They have a fundamental role during the innate immune response in activating macrophages and macrophage-like cells. HSPs participate in the cellular response to lipopolysaccharide resulting in increased inflammatory cytokine production.

HSP70 in particular, has the ability to modulate the host inflammatory immune response. The induction of pro-inflammatory cytokines by HSP70 has been shown to contribute to chronic inflammation. In contrast, HSP70 has also been reported to down regulate toll like receptors (Ferat-Osorio et al., 2014), thus inducing LPS tolerance and preventing augmentation of pro-inflammatory cytokines levels following LPS stimulation (Aneja et al., 2006).

One study showed the differential expression profiles of HSPs in AP lesions; notably, an increased expression of *DNAJC3*, *HSPA4*, *HSPA6* and *HSPB1* was detected (Goodman et al., 2014). In a case-control genetic association study with 400 individuals (183 individuals with extensive carious lesions and AP and 217 individuals with extensive carious lesions but no AP), SNPs in *HSPA1L* and *HSPA6* were shown to be associated with AP development (Maheshwari et al., 2016). Altered transmission of *HSPA1L* SNP haplotypes was also observed. SNP rs2075800 *(HSPA1L)* results in a glutamine to lysine substitution at position 602 of the protein and is predicted as probably damaging with effects on the stability and function of the encoded protein.

Other genetic studies suggested the association of persistent AP following non-surgical endodontic treatment in individuals harboring the minor allele of an *IL1B* variant (Morsani et al., 2011), meanwhile one other study did not find an association (Siqueira et al., 2009). In the latter study, allele H131 of the FcγRIIA gene and a combination of this allele with allele NA2 of the FcγRIIIB gene were suggested to be associated with persistent AP.

## Epigenetic Regulation in Pulpitis and Apical Periodontitis

Epigenetics refers to a set of mechanisms that exist above (“epi) the level of genes (Waddington, 2012). It includes a range of alterations in gene expression that occur in response to environment influences and that do not result from changes in the DNA sequence. They include chromatin remodeling, histone variants, microRNAs and long non-coding RNAs.

## Chromatin Remodeling by Histone Lys-Specific Demethylases

Epigenetic chromatin regulation has long been thought to involve permanent gene inactivation, such as X chromosome inactivation or hetero/-euchromatin, which largely involves DNA methylation and histone modifications, e.g., acetylation and methylation (Heard et al., 1997). For instance, histone 3 methylation at Lys 9 (H3K9me2/3) causes heritable chromosome inactivation by recruiting heterochromatin protein 1 (HP1), which leads to gene silencing by DNA methylation at CpG islands (Peters et al., 2002). However, an earlier study by Saccani and Natoli demonstrated for the first time that epigenetic gene regulation, by means of histone Lys-specific demethylation at H3K9me2, is a dynamic process, which regulates the expression of genes involved in the inflammatory responses (Saccani and Natoli, 2002). In this novel study, the authors showed that cultured human dendritic cells exposed to LPS expressed inflammatory cytokine genes, e.g., MDC and ELC, which occurred with transient loss of H3K9me2 epigenetic mark at the promoter regions, coincided with recruitment of RNA polymerase II and the gene expression. While the precise mechanism and the exact demethylase enzyme responsible for the loss of H3K9me2 at the cytokine genes during the inflammatory signaling need to be elucidated, this study was the first demonstration that epigenetic gene regulation by means of histone modification occurs in a dynamic fashion to control the inflammatory responses.

Dynamic role of chromatin remodeling for gene regulation was further confirmed by the discovery of histone modifying enzyme called, Lys-specific demethylase (LSD1), which is specific for H3K4 and H3K9 mono- and di-methylation (Shi et al., 2004). Subsequently, a growing list of Lys (*K*)-specific *D*e-*M*ethylases (KDMs) has been found with specific enzymatic activities against unique set of histone marks, which play specific roles in gene activation or silencing, depending on specific promoters and biological context. For instance, KDM6A and KDM6B, also known as UTX and Jmjd3, respectively, exhibit demethylase activities against their common substrate, H3K27me3, yet demonstrate different functional roles in inflammatory signaling (De Santa et al., 2009). There have been more than 30 KDMs identified, some of which have been ascribed specific roles in diverse biological functions, from embryogenesis and morphogenesis to cancer development. Detailed modes of their regulatory functions are the subject of ongoing investigations (Shi and Tsukada, 2013).

The vast majority of KDMs possess Jumanji-C domain (JmjC), which is responsible for the demethylase activity and contain co-factor housing motif for Fe(II) and α-ketoglutarate (α-KG) (Clissold and Ponting, 2001). These co-factors are required for the demethylase activity of the JmjC domain; and thus gave rise to the development of pharmaceutical inhibitors of JmjC domain-containing KDMs by use of α-KG analogs, e.g., 5-carboxyl-8-hydroxyquinoline (8-HQ) compounds and GSK-J1, which are the pan-inhibitors for the KDMs in the JmjC family (Rotili et al., 2014). Due to the regulatory roles of KDMs in wide variety of disease processes, advent of these chemical inhibitors of KDMs may offer novel opportunities for the new class of therapeutic drugs. KDMs also possess other functional domains, including the zinc finger (ZF) domain, PHD domain, and other chromodomains, e.g., “tudor” motif, which is found in proteins that associate with chromatin (Huang et al., 2006; Horton et al., 2010, 2016). ZF domain is utilized for direct DNA binding by the KDMs, as evinced in KDM2A interaction with the DNA molecules and enzymatically deplete H3K36me3, resulting in target gene suppression (Blackledge et al., 2010). KDM2A is recruited to CpG islands through its ZF domain therefore demethylates H3K36me3 preferentially in CpG islands. Since H3K36me3 is an active histone mark (He et al., 2008; Cheng et al., 2014), demethylation of H3K36 residues at the CpG islands leads to gene silencing. This is an example by which DNA methylation regulates chromatin structure through preferential interaction with specific KDMs, depending on the genetic context.

## The Role of KDMs in Epigenetic Regulation of Inflammation

The first evidence supporting the dynamic regulation of inflammation by epigenetic mechanism was demonstrated by altered enrichment of H3K9me2 along the promoter regions of pro-inflammatory cytokines, e.g., MDC and ELC, in cultured human DCs with LPS stimulation (Saccani and Natoli, 2002). However, this prior study was based on the alteration of the histone marks at the promoter regions, and the functional involvement of a specific KDM in the inflammatory responses was unknown until the discovery of KDM6B, also known as Jmjd3, as the master regulator of pro-inflammatory responses. Association between KDM6B and inflammatory signaling was found by expression screening of all known KDMs in macrophages challenged by LPS and IFN-γ (De Santa et al., 2007). Induction of KDM6B was confirmed in subsequent experiments and the functional significance was validated on gene-specific promoters by chromatin-immuno precipitation (ChIP) assay, which allows for surveying the protein-DNA interactions (De Santa et al., 2007). Furthermore, in their subsequent study, De Santa et al. demonstrated that KDM6B is recruited to more than 70% of LPS-inducible promoters by ChIP-Seq analyses, which allows for genome wide screening of the protein and target DNA interactions (De Santa et al., 2009). The vast majority of the genes with the KDM6B occupancy corresponded to the recruitment of RNA polymerase II to the promoter regions in the activated macrophages, suggesting the positive role of KDM6B in the inflammatory queue. Conversely, KDM6B knockout impaired the recruitment of RNA Pol II to the target gene promoters and the gene expression in macrophages, indicating the requirement of KDM6B in the target gene expression during inflammatory signaling (De Santa et al., 2009). In another study, KDM6B was also found to be critical for the lineage-specific differentiation of CD4 + naïve Th0 cells into various Th subsets, e.g., Th1, Th2, Th17, and the regulator T cells (Treg), through epigenetic regulation of the lineage-specific transcription factors (Li et al., 2014). For instance, CD4 + Th0 cells require expression of T-bet transcription factor for Th1 lineage differentiation and elicit pro-inflammatory immune responses, while immunomodulatory cytokines, e.g., IL-4, and GATA-3 transcription factor are required for Th0 differentiation into Th2 phenotype (Zhu et al., 2010; Kanhere et al., 2012). Th17 lineage differentiation requires the expression of RORγt transcription regulator and TGF-β/IL-6 signaling pathways, and Treg is induced by expression of FoxP3 (Kimura and Kishimoto, 2011). Ample evidence suggests the involvement of differential Th subset balance in periapical cystic and granulomatous lesions of endodontic origin. Fukada et al. demonstrated predominance of pro-inflammatory phenotype and concordant elevation of osteoclastic activities in periapical granulomas when compared with cystic lesions, by observing differential levels of lineage-specific transcription factors, e.g., T-bet, Foxp3, and GATA-3 in granulomatous and cystic lesions (Fukada et al., 2009). Furthermore, immunohistochemistry analyses of human apical granulomatous lesions revealed predominance of cells expressing IFNγ, further validating the role of Th1 subsets in the pathogenesis of endodontic lesions (Kabashima et al., 2004). As such, differential balance between CD4 + Th lineage-differentiation has a role in AP. Using a genetic approach with KDM6B ablation in T-cell specific manner, Li et al. showed loss of CD4 + Th0 differentiation into Th1 phenotype by reduced expression of T-bet transcription factor, which occurred with increased level of H3K27me2 and H3K27me3 (Li et al., 2014). Hence, these results are in keeping with the earlier report that KDM6B is an epigenetic regulator of pro-inflammatory responses through specific demethylation of H3K27me3 (De Santa et al., 2007, 2009).

Recently, regulatory effects of KDM3C were demonstrated on oral inflammatory lesions, including AP induced by pulp exposure and experimentally induced periodontitis by ligature placement (Lee et al., 2019). KDM3C is a member of JmjC-domain containing KDMs with demethylase activity specific for H3K9 moieties (Peeken et al., 2018). KDM3C ablation by gene KO led to increased inflammatory responses to oral bacterial infection, resulting in increased alveolar bone loss at the periapex or alveolar crest. Thus, KDM3C demonstrates anti-inflammatory functions.

## Mechanisms of Epigenetic Gene Regulation: Example of KDM6B and Polycomb-Group Proteins (PcGs)

**FIGURE 2:**
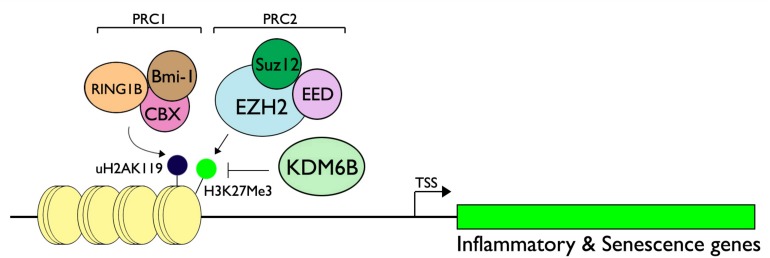
Regulation of inflammatory and senescence genes through the interplay between PcG proteins and KDM6B. Polycomb Repressive Complex (PRC) 1 is composed of Bmi-1, CBX, and RING1B, while PRC2 contains Suz12, EED, and EZH2, a methyltransferase specific for H3K27. RING1B of PRC1 demonstrates E3 ubiquitin ligase activity for H2AK119, causing monoubiquitination. When the signal is activated for pro-inflammatory cascade, e.g., bacterial infection, or due to aging, KDM6B may be upregulated and leads to demethylation of H3K27me3. This impedes the silencing activity of the PcG proteins and leads to target gene expression. Figure is revised from Kang et al. (2017).

The KDM-mediated gene regulation can be thought of a balanced activity between KDMs and their counterparts, Lys-specific methyltransferases (KMTs), which adds methyl groups on specific histone Lys residues. For instance, the methylation status of H3K27me3 is the net result of the demethylase activity of KDM6B and the methyltransferase activity of *enhancer of zeste homolog* 2 (EZH2), a component of Polycomb Repressive Complex (PRC) 2 along with other subunits, including EED and Suz12 (Cao et al., 2002; Vire et al., 2006). Enrichment of PRC2 at the target promoter regions occurs in tandem with PRC1, composed of Bmi-1, Cbx, and RING1B, which demonstrate E3 ubiquitin ligase activity on Lys 119 of H2A (Li et al., 2006). Mono-ubiquitination of H2A at Lys119 (H2AK119) then represses transcription by limiting RNA Pol II transcription elongation (Zhou et al., 2008). H3K27me3 methylation by EZH2 regulates diverse gene groups, as revealed by genome-wide ChIP-Seq analyses of H3K27me3 target promoters (Sharma et al., 2017). Mechanism of gene expression by the interplay between the PcG proteins and KDM6B is perhaps most detailed in the regulation of *INK4A* gene, which encodes p16^INK4A^ cyclin-dependent kinase (CDK) inhibitor, implicated in cellular senescence (Kang et al., 2004). During normal cell cycling, H3K27me3 enrichment is highly maintained at the *INK4A* promoter by occupancy of PRC1 and PRC2, resulting in gene silencing (Agherbi et al., 2009). Upon the onset of cellular aging, which is in part mediated through upregulation of p16^INK4A^, KDM6B level is increased and the protein is recruited to the *INK4A* promoter, causing the demethylation of H3K27me3, which then results in the loss of PCR1/2 complex enrichment at the promoter region (Agger et al., 2009). Likewise, the ubiquitinated H2AK119 limited the expression of diverse chemokines and inflammatory mediators, e.g., CXCLl1/MIP2, CCL5/Rantes, and CXCL10/IP-10, suggesting the suppressive effect of PcG silencing on inflammatory gene expression (Zhou et al., 2008). Thus, the PcG-mediated gene silencing requires both H3K27me3 methylation and H2AK119 ubiquitination, resulting in chromatin condensation and gene silencing, which is reversed by upregulation of KDM6B upon the environmental or physiological queue (Figure 2). On the contrary, a recent report showed that EZH2 may be necessary for the pro-inflammatory signaling, and this was demonstrated in cultured human dental pulp cells (HDP) and animal models. Hui et al. showed that silencing of EZH2 in cultured HDPs attenuated the secretion of inflammatory cytokines, e.g., IL-6, IL-8, IL-15, CCL2, and CXCL12 (Hui et al., 2014). Using a rat pulpitis model, the authors also showed reduction of pulpitis responses in the animals exposed to DZNep, a pharmacological inhibitor of EZH2, further demonstrating the physiological role of EZH2 in pro-inflammatory signaling in dental pulp. Therefore, the role of PcG-mediated gene silencing and activation by KDMs may be context-dependent and will require further research to elucidate the precise mechanism during oral inflammatory signaling.

## The Role of MicroRNAs in Regulating Pulpal and Periapical Disease

MicroRNAs, small non-coding RNA molecules, regulate gene expression by inhibiting protein translation or by degrading the messenger RNA transcript. They play a critical role in normal cellular function and according to bioinformatics predictions, they regulate at least one third of all messenger RNAs. While microRNAs regulate both the innate and adaptive immune response, their role in regulating endodontic disease is only just beginning to be explored. Exploration of the differential expression of microRNAs in pulpal and periapical disease were reported a few years ago (Zhong et al., 2012; Chan et al., 2013). Initially, an observational study was done in which inflamed pulp from carious teeth diagnosed with symptomatic or asymptomatic irreversible pulpitis were compared to pulp from healthy teeth. As compared to normal pulps, 3 microRNAs were upregulated, and 33 microRNAs were downregulated in inflamed pulps. A similar observational study was conducted in diseased periapical tissues (Chan et al., 2013). Twenty-four microRNAs were downregulated as compared to normal controls. These included some of the same microRNAs downregulated in pulpitis. Down-regulation of microRNAs results in an increase in their respective target messenger RNAs as microRNAs are negative regulators In contrast, a similar study on periapical lesion found that miR-155 was upregulated (Yue et al., 2016). This microRNA targets semaphorin 3a, known to play a role in bone remodeling and macrophage apoptosis. Another microRNA reported to have a potential role in the pathogenesis of AP is miR-335-5p, a promoter of RANKL (Yue et al., 2017).

Several members of the microRNA 181 family were downregulated in both inflamed pulps and in inflamed periapical diseases. These include miR-181a, known to regulate expression of toll-like receptor 4, which plays an important role in pathogen recognition and activation of the innate immune response. To further examine the role of the miR-181a in the TLR agonist-induced response in pulpitis an *in vitro* model of human pulp fibroblasts was used (Galicia et al., 2014). These cells recognize conserved microbial patterns through TLRs. Stimulation of human pulp fibroblasts with LPS from *Porphyromonas gingivalis* (*Pg*) resulted in downregulation of miR-181a and increased expression of IL-8. A miR-181a binding site on the 3′UTR of IL-8 was identified by *in silico* analysis. This was confirmed by dual-luciferase assays. Taken together the data demonstrate that human pulp fibroblasts respond to *Pg* LPS by downregulating expression of miR-181a, which in turn increases expression of the powerful chemoattractant IL-8.

**FIGURE 3:**
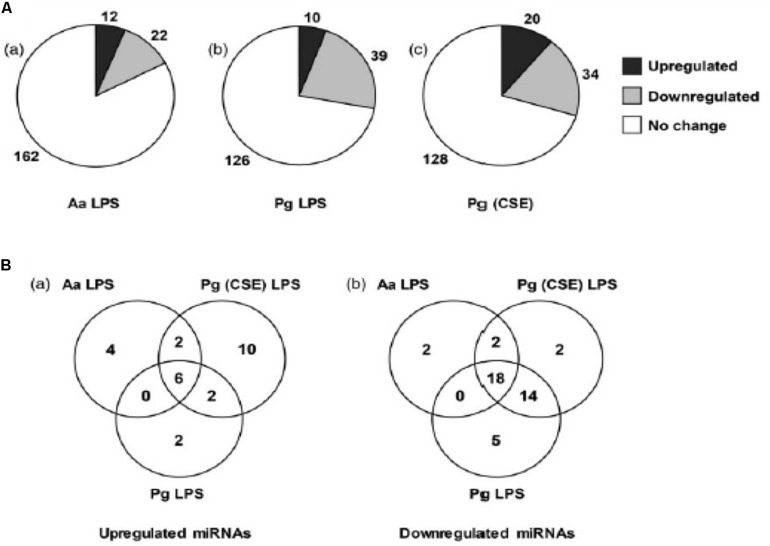
LPS induced differential expression of microRNAs in THP-1 differentiated human macrophages. **(A)** RNA extracted from macrophages treated with LPS from *A. actinomycetemcomitans (Aa)*, *P. gingivalis (Pg)* and *P. gingivalis* grown in cigarette smoke extract (Pg [CSE]) was profiled form microRNAs using NanoString technologies. **(B)** Venn diagram showing convergent and divergent microRNA profile in response to LPS treatment. Reproduced with permission from Naqvi et al. (2014).

Macrophages also play an important role in the innate immune response of the pulp and periapical tissues. An *in vitro* model was used to study the macrophage microRNA response to LPS. The microRNA expression pattern in CD14 + human macrophages challenged with *E. coli* LPS changes in a time and dose–dependent manner (Naqvi et al., 2016). The LPS responsive microRNAs target key signaling and pathogen recognition pathways including PIK3-Akt, MAPK, ErbB, Wnt and TGF-β. A similar study compared the microRNA profiles in human macrophages challenged with LPS from 3 different sources- *Aggregatibacter actinomycetemcomitans* (*Aa*), *Pg* and *Pg* grown in cigarette smoke extract (Naqvi et al., 2014). A “core” microRNA response was noted with 24 microRNAs being differentially expressed across all 3 LPS species (Figure 3). At the same time, LPS specific responses were noted (Figure 3). This demonstrates that environmentally induced alterations in LPS (such as cigarette smoking) modify the miRNA response.

Most studies on microRNAs have focused on host microRNAs. Viruses, especially those with DNA genome, encode miRNAs in order to regulate their life cycle inside the host (Ambros, 2004). Host proteins recognize the viral transcripts generated in the nucleus and process them in a way similar to the canonical host microRNA pathway. To date, more than 200 different microRNAs of viral origin have been identified. Viral microRNAs may regulate both viral and/or host transcripts. Given the capacity to regulate hundreds of probable host transcripts, viral microRNAs have the potential to significantly influence the host transcriptome. An observational study was done to compare the viral encoded microRNA profiles in healthy and diseased human pulps (Naqvi et al., 2016). Four viral microRNAs- HSV1-miR-H1, HCMV-miR-US4, HCMV-miR-UL70-3p, and KSHV-miR-K12-3 were expressed at higher levels in diseased human dental pulps. *In silico* target prediction of the differentially expressed viral microRNAs were then used to identify the potential host target genes that are important in defense against pathogens. The target genes were key mediators involved in the detection of microbial ligands (TLR-1), proteolysis (MMP24), chemotaxis (Chemokine ligand 25), pro- and anti-inflammatory cytokines (IL8, IL-10) and, signal transduction molecules (Inducible T cell co-stimulator). This suggests that miRNAs may play a role in interspecies regulation of pulpal health and disease. Further research is needed to elucidate the mechanisms by which these viral miRNAs can potentially modulate the host response.

## Conclusion

Recent evidence shows that that gene expression, gene-gene interactions, as well as epigenetic alterations may contribute to the etiology of pulpitis and AP. Understanding the underlying molecular mechanisms in pulpitis and AP may provide insights into the development of improved diagnostic tools of the dental pulp status, prediction of dental pulp survival and personalized treatment strategies. In addition to removal of etiologic factors, future therapeutic approaches may focus on localized downregulation of proinflammatory signals, and upregulation of regenerative mechanisms.

## Author Contributions

AF conceived idea of manuscript, contributed to content, and did overall editing and submission. AK contributed to content and overall editing. RS and MK contributed to content.

## Conflict of Interest

The authors declare that the research was conducted in the absence of any commercial or financial relationships that could be construed as a potential conflict of interest.
